# Valvular Cardiomyopathy in Aortic Valve Regurgitation Correlates with Myocardial Fibrosis

**DOI:** 10.3390/jcm12082915

**Published:** 2023-04-17

**Authors:** Johannes Petersen, Shahria Iqbal, Naomi Gedeon, Benjamin Kloth, Simon Pecha, Yalin Yildirim, Thomas Eschenhagen, Hermann Reichenspurner, Torsten Christ, Evaldas Girdauskas

**Affiliations:** 1 Department of Cardiovascular Surgery, University Heart and Vascular Center Hamburg, Martinistraße 52, 20246 Hamburg, Germany; 2 DZHK (German Centre for Cardiovascular Research), Partner Site Hamburg/Kiel/Lübeck, 20246 Hamburg, Germany; 3 Institute of Experimental Pharmacology and Toxicology, University Medical Center Hamburg-Eppendorf, 20246 Hamburg, Germany; 4 Department of Cardiothoracic Surgery, Augsburg University Hospital, 86156 Augsburg, Germany

**Keywords:** valvular cardiomyopathy, myocardial fibrosis, aortic valve disease, aortic regurgitation

## Abstract

**Objective:** At the tissue level, disruption of the extracellular matrix network leads to irreversible cardiac fibrosis, which contributes to myocardial dysfunction. At the myocyte level, downregulation of beta-adrenoceptors (beta-AR) reduces adaptation to increased workload. The aim of our study was to analyse the correlation between myocardial fibrosis and beta-AR sensitivity in patients with aortic valve (AV) disease. **Methods:** A total of 92 consecutive patients who underwent elective AV surgery between 2017–2019 were included in our study (51 with aortic regurgitation (AR-group); 41 with aortic stenosis (AS-group) and left ventricular (LV) biopsies were obtained intraoperatively. In vitro force contractility testing was performed by measuring beta-AR sensitivity (−log EC_50_[ISO]). In parallel, a quantitative analysis of myocardial fibrosis burden was performed. **Results:** Mean age at the time of AV surgery was not statistically different in both groups (AR: 53.3 ± 15.3 years vs. AS: 58.7 ± 17.0 years; *p* = 0.116). The LV end-diastolic diameter was significantly enlarged in the AR-group when compared to the AS-group (59.4 ± 15.6 vs. 39.7 ± 21.2; *p* < 0.001). Analysis of beta-AR sensitivity (AR: −6.769 vs. AS: −6.659; *p* = 0.316) and myocardial fibrosis (AR: 8.9% vs. AS: 11.3%; *p* = 0.284) showed no significant differences between patients with AS and AR. There was no correlation between myocardial fibrosis and beta-AR sensitivity in the whole study cohort (R = 0.1987; *p* = 0.100) or in the AS-subgroup (R = 0.009; *p* = 0.960). However, significant correlation of fibrosis and beta-AR sensitivity was seen in AR-patients (R = 0.363; *p* = 0.023). **Conclusion:** More severe myocardial fibrosis was associated with reduced beta-AR sensitivity in patients presenting with AR but not with AS. Therefore, our results suggest that in patients with AR, cellular myocardial dysfunction is present and correlates with the extent of myocardial fibrosis in the myocardium.

## 1. Introduction

Aortic valve (AV) surgery is the treatment of choice in symptomatic patients with severe aortic valve stenosis (AS)/aortic valve regurgitation (AR), and in asymptomatic patients with impaired left ventricular function or enlarged left ventricular end-systolic diameter [1]. Severe aortic valve disease can lead to a so-called valvular cardiomyopathy, which occurs even before the deterioration of ventricular function and the onset of symptoms. Death and heart failure in patients with chronic severe aortic valve disease is not primarily a result of the deformed valve, but from abnormal myocardial burden. In particular, AR patients have an enlarged left ventricle prior to the onset of heart failure symptoms and if severe aortic valve disease remains untreated, progressive heart failure occurs [2]. Therefore, timely AV replacement or repair is the only treatment option for such patients. However, heart failure often progresses despite successful AV surgery, since in AR patients, severe left ventricular dysfunction (e.g., LVEF ≤ 30%) may persist or even deteriorate after surgery and impacts significantly a reduced long-term survival [3].

However, the reasons of postoperative LV deterioration are still unknown. Our study group was able to show that in the asymptomatic stage of AV disease with simultaneously enlarged LVEDD, a significant cellular myocardial dysfunction, measured by in vitro contractility, is already present [4]. A decreased responsiveness of beta-adrenoceptors (beta-AR) at the myocyte level could be an expression of the adaptation of increased workload in severe AV disease. At the tissue level, a disruption of the extracellular matrix network leads to irreversible cardiac fibrosis, which is a restrictive part of LV remodelling and contributes to myocardial dysfunction. In animal experiments, it was shown that severe AR is linked to myocardial fibrosis and necrosis without inflammation, resulting in congestive heart failure [5]. Such abnormal extracellular matrix (ECM) produced by cardiac fibroblast (e.g., non-collagen ECM such as fibronectin) are a primary response to the mechanical burden due to the severe AR [2]. Myocardial fibrosis, but not myocyte beta-AR function, can be measured in vivo.

Therefore, the aim of our study was to analyse whether a correlation between myocardial fibrosis and in vitro beta-AR sensitivity in patients with aortic valve disease may exist.

## 2. Methods

### 2.1. Ethical Statement

The investigation conforms to the principles outlined in the Declaration of Helsinki and patients were included after written informed consent. Our local ethics committee of the “Ärztekammer Hamburg” approved the study protocol in 21 February 2016 (No. PV3759).

### 2.2. Surgical Intervention

The study group consists of previously described patient group which underwent elective AV surgery at our institution between February 2017 and November 2019 [2]. One left ventricular myocardial sample was obtained during open AV surgery by resection of a small sample (1–5 mm) from the left ventricular outflow tract. The majority of AV surgery was performed through a partial upper mini-sternotomy. After establishment of cardiopulmonary bypass mild systemic hypothermia of 33–34 °C was implemented, and the heart was arrested by a single antegrade shot of crystalloid Custodiol (Dr. Koehler, Bensheim, Germany) cardioplegic solution. Standard AV replacement was performed in patients with AS, while most patients with AR underwent AV repair surgery.

Myocardial strips (5–10 mm) were used for histological analysis (n = 92 patients) and served as our primary study group. Both groups were divided according to their AV disease, resulting in 51 patients with AR (**AR-group**) and 41 patients with AS (**AS-group**).

### 2.3. Histology

**Figure 1:**
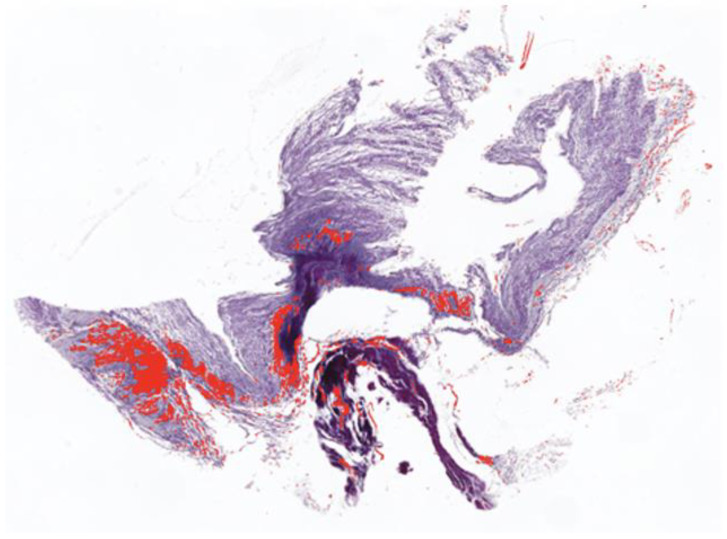
Histological section of a patient with aortic valve regurgitation and quantification of fibrosis.

Myocardial samples of all 92 patients were fixed in 4% paraformaldehyde solution for 24 h. Afterwards, tissues were embedded in paraffin and serial 5 µM sections were made with Leica microtome and mounted onto labelled slides. PicroSirius Red staining (connective tissue stain) was done to evaluate fibrosis in paraffin-embedded heart sections. Quantification of myocardial fibrosis volume was calculated as the ratio of the area positively stained for fibrosis compared to the total myocardial area (Figure 1). Analysis was performed with Image J [6] by three different independent researchers (J.P.; S.I.; N.G.). To a maximum of four sections were analysed per patient and a mean value was calculated (in percentage).

### 2.4. In Vitro Contractility

The method of in vitro contractility has been prescribed previously [4]. Briefly, the small LV myocardial sample was immediately placed into a non-oxygenated cardioplegic solution [in mM: NaCl 100, taurine 50, glucose 20, KCl 10, MgSO4 5, MOPS (3-(N-morpholino)propanesulfonic acid) 5, KH2PO4 1.2) containing 30 mM of the myosin ATPase inhibitor BDM (2,3-butanedione monoxime) at room temperature.

The myocardial tissue strips were used for contractility experiments. Experiments were performed in modified Tyrode’s solution containing (mM): NaCl 126.7, KCl 5.4, CaCl2 1.8, MgCl2 1.05, NaH2PO4 0.42, NaHCO3 22, EDTA 0.04, ascorbic acid 0.2 and glucose 5.0. The solution was maintained at pH 7.4 by bubbling with a mixture of 5% CO_2_ and 95% O_2_. Strips were attached to SWEMA 4–45 strain gauge transducers in an apparatus containing above solution at 37 °C and paced at 1 Hz. Myocardial samples were pre-stretched to 50% of the resting tension associated with maximum developed force. In all experiments, we followed a protocol aimed to minimize the effects of endogenous catecholamines by using 5 mM phenoxybenzamine for 90 min [4]. Force was recorded using Chart Pro for Windows version 5.51 analysis programme (ADI Instruments, Castle Hill, Australia). Isoprenaline (ISO) (0.1 nmol to 30 µmol) was used for incremental beta-AR stimulation followed by an application of forskolin (10 µmol), which activates adenylyl cyclase directly. Maximum force was evoked by the subsequent addition of a high concentration of calcium (8 mM). Isometric contractions were measured as contractile force in mN. Beta-AR sensitivity was defined by logEC_50_ [ISO], which is the concentration of ISO required to reach 50% maximal ISO-induced increase in force. As a measure of maximum effect size of beta-AR mediated inotropy, we calculated the fraction of maximum inotropic response induced by 30 mmol ISO compared to the inotropic response in the presence of high calcium (8 mM), expressed as F_ISO/Ca_ (%) [4].

### 2.5. Statistical Analysis

Continuous variables are presented as mean ± standard deviation; categorical variables are expressed as absolute and relative frequencies. A univariate linear regressions model was used to analyse the impact of fibrosis on logEC_50_ [ISO] and on F_ISO/Ca_. Double-sided *p*-values smaller than 0.05 were considered statistically significant. All statistical analyses were accomplished with the IBM SPSS 23 software (IBM Corp., New York, NY, USA).

## 3. Results

### 3.1. Study Population

Patient characteristics are outlined in Table 1. Mean age at the time of index AV surgery was similar in both groups (AR: 53.3 ± 15.3 years vs. AS: 58.7 ± 17.0 years; *p* = 0.116). AR-patients were more frequently male (80.4% vs. 39.0%; *p* < 0.001), had a significant larger LV end-diastolic diameter (59.4 ± 15.6 vs. 39.7 ± 21.2 mm; *p* < 0.001) and a significant lower LV ejection fraction (60.5 ± 7.1 vs. 52.5 ± 9.9%; *p* < 0.001). Congenital AV disease (unicuspid and bicuspid aortic valve) was present in 54.9% of the AR patients compared to 48.8% of the AS patients (*p* = 0.559). Body mass index was significantly lower in the AR-group (*p* = 0.022). Further cardiovascular risk factors and comorbidities did not differ between both groups (e.g., arterial hypertension, dyslipidemia, coronary artery disease). NYHA class II or more was present 49% of the AR patients and in 75% of the AS patients (*p* = 0.002). More patients with AR received Betablocker compared to AS (45% vs. 29% *p* = 0.169) and ACE-inhibitors (34% vs. 47%; *p* = 0.102), yet both not with statistical difference) Table 2.

### 3.2. Myocardial Fibrosis

The mean incidence of myocardial fibrosis in the whole study group was 9.9% (range: 0.3–52.0%) without a statistical difference between aortic stenosis and regurgitation (AR: 8.9% vs. AS: 11.3%; *p* = 0.284). In the whole study group, there was no significant correlation between myocardial fibrosis and age (R = 0.040; *p* = 0.740), LV ejection fraction (R = 0.031; *p* = 0.797), as well as all other clinical parameters. In the AR-group, significant correlations were identified between myocardial fibrosis and hyperlipidemia (R = 0.395; *p* = 0.013) and arterial hypertension (R = 0.360; *p* = 0.024), whereas in the AS-group, no significant correlation was seen (hyperlipidemia: R = −0.208; *p* = 0.261; arterial hypertension: R = −0.198; *p* = 0.285).

### 3.3. In Vitro Contractility and Myocardial Fibrosis

**Figure 2:**
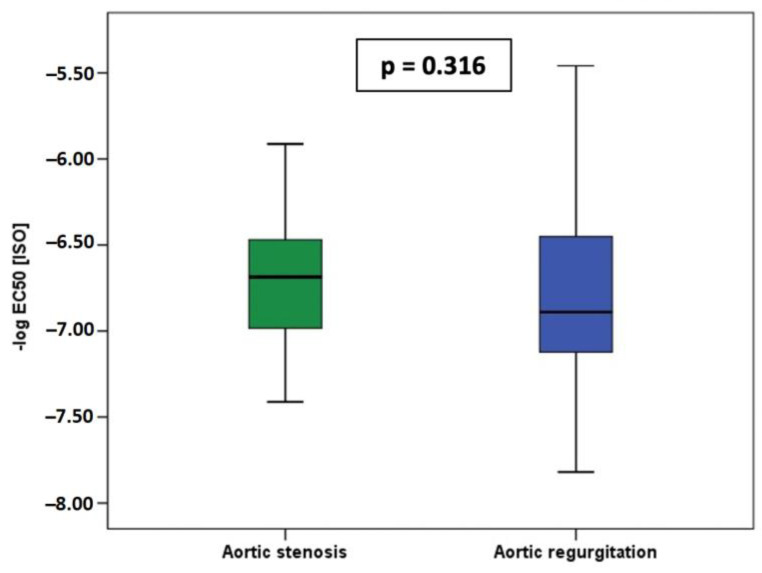
No significant differences of Beta-AR sensitivity (−log EC_50_[ISO]) between patients with aortic stenosis (green) and regurgitation (blue).

**Figure 3:**
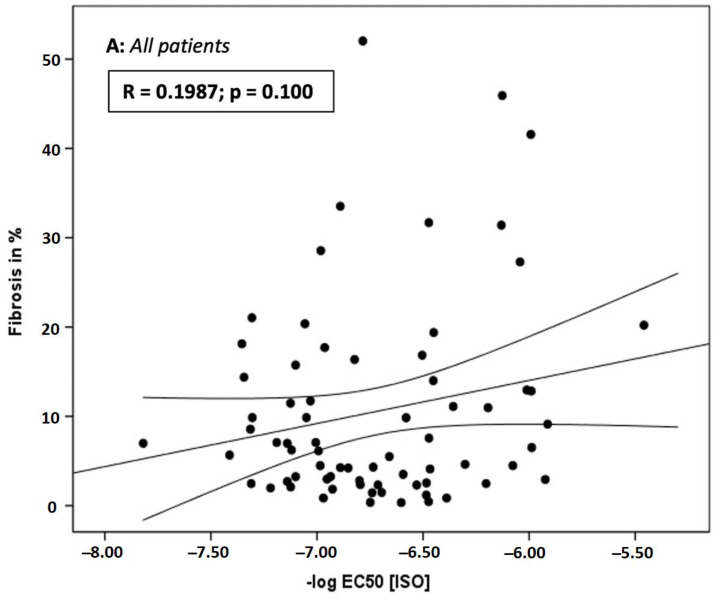

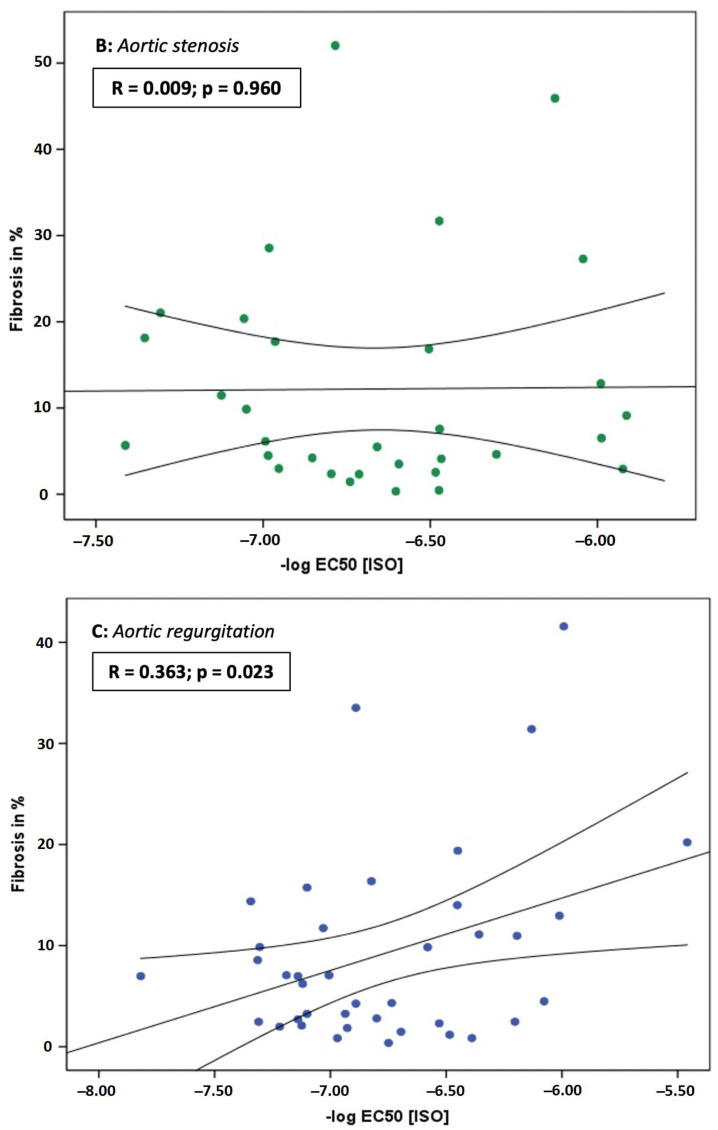
Correlation of fibrosis (in%) with Beta-AR sensitivity (−log EC_50_[ISO]) in the whole study cohort (n = 93) (**A**) and in subgroups with stenosis (**B**, green) and regurgitation (**C**, blue).

Analysis of beta-AR sensitivity (−log EC_50_[ISO]) (AR: −6.769 vs. AS: −6.659; *p* = 0.316) showed no significant differences between patients with aortic stenosis and regurgitation (Figure 2). There was no correlation of fibrosis (in%) with beta-AR sensitivity (-log EC_50_[ISO]) in the whole study cohort (R = 0.1987; *p* = 0.100; Figure 3A) or in the AS-subgroup (R = 0.009; *p* = 0.960; Figure 3B). However, a significant correlation between myocardial fibrosis and in vitro beta-AR sensitivity was seen in patients with aortic regurgitation (R = 0.363; *p* = 0.023) (Figure 3C).

Maximum inotropic response measured by F_ISO/_Ca was significantly higher in AR patients (AR: 70.9 ± 20.3 vs. AS: 58.2 ± 21.4; *p* = 0.012). No correlation of myocardial fibrosis with F_ISO_/Ca was seen in the whole study group (R = −0.153; *p* = 0.202), or in the AS- (R = −0.077; *p* = 0.675) or AR-subgroups (R = −0.173; *p* = 0.293).

## 4. Discussion

Our work demonstrated that in human myocardial tissue of patients with severe aortic valve disease, myocardial fibrosis is present but without a significant difference in AR and AS. However, a significant correlation of fibrosis and in vitro beta-AR sensitivity was seen in patients with AR.

Overall, severe aortic valve disease results in morphological changes of the left ventricle (e.g., hypertrophy) which often do not correlate with the clinical presentation of the patients [4,7,8]. During the stage of compensatory hypertrophy especially, an extension of elastic collagen in the myocardium is present, in order to maintain contractility [9].

Myocardial fibrosis in AS has been studied extensively. Azevedo was able to show an association between a high degree of fibrosis with an increased mortality risk after AV surgery in AS patients [10]. Most recently, an MRI study confirmed the negative impact of myocardial fibrosis measured by extracellular volume fraction [11]. Furthermore, the extent of myocardial fibrosis correlated with the symptoms (e.g., NYHA class) and the myocardial fibrosis was not reversible after AV surgery in symptomatic AS patients [12]. On the contrary, in a recent study of 61 asymptomatic patients, AV intervention resulted in a reduction of diffuse fibrosis [13]. Such findings emphasize the importance of assessment of myocardial fibrosis (e.g., by MRI) in the risk stratification of asymptomatic AS patients [14]. In addition, our study group was able to show that in the asymptomatic stage of AV disease with simultaneously enlarged LVEDD, a significant cellular myocardial dysfunction, measured by in vitro contractility, is already present [4].

In chronic AR, only limited data regarding myocardial fibrosis and contractility is available. A small group of patients (n = 28) with chronic AR showed a smaller percentage of myocardial fibrosis (3.5%) compared to our studied AR patients (8.9%). Furthermore, no correlation between fibrosis and left ventricular function measured by echocardiography was detected by Elias et al. [15]. However, the 84% of the studied patients presented primarily with rheumatic disease and therefore no final conclusion can be drawn by this different group of patients. In contrast, a study with 14 AR patients identified increased myocardial fibrosis (≥10%) in patients at an early stage of eccentric hypertrophy, [16] suggesting early non-physiologic changes at the cardiomyocyte level in valvular cardiomyopathy. Most recently, an MRI study suggested that myocardial scarring in AR patients was associated with a 2.5-fold increase in risk of mortality, which could be reduced by aortic valve surgery instead of medical therapy [17]. Patients who undergo coronary artery bypass grafting (CABG) may also exhibit myocardial fibrosis, similar to AV disease. A biopsy study of patients who had CABG surgery found that the amount of tissue was lower than in our study, ranging from 0.7% to 4.0% [18]. However, this study group only took a small biopsy of a selected area without any macroscopic pathology, making it difficult to compare with our obtained tissue extracted from the left ventricular outflow graft. Nonetheless, an MRI study has shown that the presence of myocardial fibrosis in patients with coronary artery disease increases the risk of sudden death, emphasizing its impact on the outcomes of patients with cardiomyopathy [19]. Such findings go hand in hand with our results, demonstrating that an increase in myocardial fibrosis is associated with a decreased beta-AR sensitivity, resulting in a possible worse outcome. Our described differences of in vitro contractility and myocardial fibrosis between AS and AR can be explained by the pathomechanism of both diseases. In patients with AS, there is a presence of concentric hypertrophy resulting in hypertrophy of the cardiomyocytes. These hypertrophied cardiomyocytes are accustomed to the increased afterload caused by severe AS, and thus, myocardial fibrosis does not significantly affect their in vitro contractility. However, in AR patients, eccentric dilatation occurs, and thus, myocardial fibrosis plays a more significant role, leading to reduced in vitro contractility. Future follow-up studies should clarify the impact on long-term prognosis after AV surgery.

### 4.1. Clinical Perspective

The identification of in vitro myocardial dysfunction in mostly asymptomatic patients in our, and previous, studies [4] highlights the progressive left ventricular remodelling at a cellular and tissue level in patients with AV disease and valvular cardiomyopathy. Our study confirmed an increase in myocardial fibrosis in patients with AR correlating with a reduced beta-AR sensitivity. Such finding underlines, that there are still missing pieces for decision-making regarding the most appropriate time of surgery in asymptomatic patients with severe AR. Timing of surgery in asymptomatic patients is difficult since the current ESC/EACTS guidelines recommend surgery only when left ventricular remodelling has already started (LVEF ≤ 50%; LVESD > 50 mm or LVESD > 25 mm/m^2^ BSA) [1]. In case of asymptomatic severe AV disease with normal left ventricular function a so-called watchful-waiting strategy is preferred at the moment. However, in some patients the timing of surgery when the symptoms appear is too late since left ventricular deterioration has already started. Mentias et al. showed convincingly in an observational study that 96% of the deaths occurred in patients with severe AR and preserved LV function and a normal indexed LVESD of less than 25 mm/m^2^ [20]. Such findings underline the importance of right patient-selection for early AV intervention. For this reason, the potential role of different biomarkers needs further attention, in order to guide the watchful-waiting strategy. Therefore, our present study provides a precise cornerstone which can be used for validation of a circulating (e.g., proBNP, galectin-3, microRNAs) or imaging (e.g., left ventricular global longitudinal strain in echocardiography or T1-mapping in magnetic resonance imaging technique) biomarker for detection of LV dysfunction before it will become irreversible. This could help to provide an additional decision-making aid in the timing of the surgery in order to prevent postoperative persistence of reduced cardiac.

### 4.2. Limitations

Our study analysed only cellular and tissue-related changes in patients with AV disease. This approach enables us only to describe the in vitro changes in valvular cardiomyopathy. However, pathological signal cascades have not been studied by our in vitro approach and future studies (e.g., protein and gene expression analysis) might help to link the ventricular remodelling to a pathological cascade on the cellular level. Moreover, the results of our analysis might be biased by a potential sampling error. Some segments of the LV might have more pronounced remodelling than others. However, many other studies also primarily work with ventricular samples only obtained from one location of the myocardium. In addition, a follow-up of the study group has not been performed so that the clinical impact of our in vitro findings is not verified so far. However, the main goal of our study was to identify differences of in vitro contractility and fibrosis in patients with aortic valve disease and a follow-up study is planned in the future.

## 5. Conclusions

Myocardial fibrosis exists in patients with aortic valve disease. In patients with aortic regurgitation especially, cellular myocardial dysfunction is present and correlates with the extent of fibrosis in the myocardium. Our findings could help to provide an additional decision-making aid in the timing of the surgery before reduced cardiac function persists.

## Figures and Tables

**Table 1:** Patient characteristics of patients with aortic stenosis (n = 41) and regurgitation (n = 51).

	Aortic Stenosis (AS) (n = 41)	Aortic Regurgitation (AR) (n = 51)	*p*-Value
Age (years)	58.7 ± 17.0	53.3 ± 15.3	0.116
Male	16 (39.0%)	41 (80.4%)	**<0.001**
Body mass index (kg/m^2^)	28.2 ± 5.0	26.0 ± 3.9	**0.022**
Arterial hypertension	21 (51.1%)	20 (46.0%)	0.540
Dyslipidemia	11 (27.5%)	11 (22.0%)	0.546
Coronary artery disease	10 (25.0%)	6 (12.0%)	0.109
Diabetes mellitus	5 (12.2%)	1 (1.9%)	0.103
Chronic kidney disease (GFR < 60 mL/min)	1 (2.4%)	3 (5.9%)	0.199
Atrial fibrillation	3 (7.3%)	4 (7.8%)	0.462
NYHA			**0.002**
- Class II	13 (32.5%)	19 (37.3%)	
- Class III	17 (42.4%)	4 (7.8%)	
- Class IV	0	2 (3.9%)	
Left ventricular (LV) ejection fraction (%)	60.5 ± 7.1	52.5 ± 9.9	**<0.001**
LV end-diastolic diameter (mm)	39.7 ± 21.2	59.4 ± 15.6	**<0.001**
Aortic valve morphology			**0.007**
- Tricuspid	20 (48.8%)	21 (41.2%)	
- Bicuspid	12 (29.3%)	28 (54.9%)	
- Unicuspid	9 (22.0%)	2 (3.9%)	
Preoperative pro-BNP value (pg/mL)	860.6 (39–3696)	1045.1 (24–9334)	0.646
Medication with Betablocker	11 (27.5%)	20 (40.0%)	0.215

**Table 2:** Preoperative medication of patients with aortic stenosis (n = 41) and regurgitation (n = 51).

	Aortic Stenosis (AS) (n = 41)	Aortic Regurgitation (AR) (n = 51)	*p*-Value
Medication with Betablocker	11 (27.5%)	20 (40.0%)	0.215
ACE inhibitor	14 (34.1%)	24 (47.0%)	0.102
AT-II receptor blocker	6 (14.6%)	8 (15.6%)	0.978
Ca-channel blocker	12 (29.2%)	7 (13.7%)	0.107
Statin	9 (21.9%)	8 (15.6%)	0.482

## Data Availability

The raw data supporting the conclusions of this article will be made available by the authors, without undue reservation. The corresponding author, Johannes Petersen (joh.petersen@uke.de), can be contacted for a request of the data.
